# The Sum of the Leg Length Discrepancy and the Difference in Global Femoral Offset Is Equal to That of the Contralateral Intact Side and Improves Postoperative Outcomes after Total Hip Arthroplasty: A Three-Dimensional Analysis

**DOI:** 10.3390/jcm13061698

**Published:** 2024-03-15

**Authors:** Norio Imai, Yuki Hirano, Yuki Endo, Yoji Horigome, Hayato Suzuki, Hiroyuki Kawashima

**Affiliations:** 1 Division of Comprehensive Musculoskeletal Medicine, Niigata University Graduate School of Medical and Dental Sciences, Niigata City 951-8510, Japan; 2 Division of Orthopedic Surgery, Department of Regenerative and Transplant Medicine, Niigata University Graduate School of Medical and Dental Sciences, Niigata City 951-8510, Japan

**Keywords:** total hip arthroplasty, global femoral offset, leg length discrepancy, Harris Hip Score, outcome

## Abstract

**Background/Objectives**: Global femoral offset (GFO) and leg length discrepancy (LLD) affect outcomes after total hip arthroplasty (THA). Moreover, the sum of the difference in GFO between the THA and non-surgical sides and LLD (SGL) reportedly affects the outcomes in a two-dimensional evaluation. We examined the association of the GFO, LLD, and SGL with the Harris Hip Score (HHS) using a three-dimensional (3D) evaluation. **Methods**: We retrospectively surveyed 172 patients with hemilateral hip osteoarthritis who underwent THA. The GFO, LLD, and SGL were measured using the 3D pelvis and femur models; these models were adjusted for the pelvis and femur, and the coordinate systems were parallelized. Furthermore, their relationship with the modified HHS (mHHS) 1 year after THA was determined. **Results**: Significant correlations were found among mHHS, GFO, and SGL in the binomial group, whereas LLD was not significantly correlated. The optimal values of GFO and SGL were 1.01 mm and 0.18 mm/100 cm body height, respectively, which were considered optimal when the SGL values were approximately equal to those of the non-operative side. The optimal ranges for GFO and SGL were −1.65 to 3.67 mm and −4.78 to 5.14 mm/100 cm, respectively. **Conclusions**: Our findings were obtained after adjusting the pelvis and femur to a unified coordinate system. Therefore, the results of this study can be directly applied to 3D planning.

## 1. Introduction

Total hip arthroplasty (THA) is a well-known procedure for relieving hip arthritis-induced pain and improving hip functions, such as walking and activities of daily living. Previously, several parameters were described to improve the hip function, the femoral offset (FO) [the distance between the femoral head center and the axis of the proximal femur], and global FO (GFO), which is the sum of FO and acetabular offset (AO), which is the distance between the teardrop and the femoral head center affect outcomes (such as hip function and activities of daily living) after THA [1,2,3,4,5,6]. Notably, most outcomes reported to date are based on measurements using plain radiographs, where the FO is influenced by the internal and external rotation of the hip joint [1,2,3,4,5], except for the report by Hirano et al. [6], which used a three-dimensional (3D) method.

Leg length differences also affect hip function and postoperative outcomes, such as walking speed and stride length after THA [7,8,9,10]. A two-dimensional (2D) study using computed tomography (CT)-derived images previously described a relationship between the absolute value of the sum of leg length discrepancy (LLD), FO, and AO compared with the non-surgical side; a smaller absolute value of the sum may consequently be recommended to restore an absolute value of the sum; reduce the postoperative trochanteric pain syndrome; and improve the clinical outcome evaluated using the Harris Hip Score (HHS), Hip Disability and Osteoarthritis Outcome Score, and the EuroQol of patients after THA [11]. Employing evaluations obtained using 2D techniques to plan the implantation angle is difficult; therefore, including the anteversion angle of the stem before THA is necessary. Similarly, using a unified coordinate system is essential because plain radiographs cannot assess the anteroposterior femoral offset (AFO).

Therefore, this study aimed to examine the association of LLD, GFO, and the sum of the difference in GFO between the THA and non-surgical sides and LLD (SGL) with HHS. Based on these measurements, we also determined the optimal offset and/or LLD and its range to improve postoperative outcomes after THA. As in a previous report [11], we hypothesized that outcomes might be better when the LLD is similar, and GFO is slightly greater on the THA side [6].

## 2. Materials and Methods

### 2.1. Patients

Patients who underwent THA at our institution between 1 January 2010 and 31 December 2021 were enrolled in this study. During the study period, 534 THAs were performed, including patients with symptomatic hemilateral secondary hip osteoarthritis (HOA) with asymptomatic and a center-to-edge angle of ≥25° at the non-operative hip. Patients with a history of surgery in the lumbar spine, knee, or lower leg, such as severe knee OA or those with severe developmental dysplasia of the hip joint evaluated as Crowe type III or IV, were excluded. We also excluded patients with psychiatric and neurologic disorders. In total, 172 patients (131 females and 41 males) were enrolled in this survey. Notably, all THAs were performed by seven experienced orthopedic surgeons using the anterolateral supine approach [12,13,14,15,16]. The acetabular component was used to restore the original hip center and was planned relative to the functional pelvic plane (FPP), the sagittal tilt of the anterior pelvic plane (APP), and the plane, which included the most anterior part of the pubic symphysis and the anterior superior iliac spines of both sides, with 40° radiographic inclination and 15° radiographic anteversion [17]. It was placed using a mechanical guide [18] or CT-based navigation [19], according to the pre-operative planning; the accuracy of the implantation was described within 3° in both devices [18,19]. The stem was inserted at an angle of 20–25° at the anteversion relative to the posterior condylar plane, containing the most posterior ends of the bilateral femoral condyles and greater trochanter [20]. It was applied to the shape of the femoral shaft’s medullary canal to fit the medial and lateral femoral shafts aimed to restore the offset and LLD and adjusted using the combined anteversion theory [21,22]. The LLD value was planned to be equal to that of the non-operated intact side. During the study period, we did not use an offset and elevated-rim liner. However, a ceramic head with a diameter of 32 or 36 mm was used.

### 2.2. Measurement

We reconstructed the 3D pelvis and femur models and evaluated the implant positioning using ZedView^®^ software (version 16.0; Lexi, Tokyo, Japan) from the CT images taken for 3D planning pre-operatively and 1 week postoperatively [19,23]. Approximately 600 sliced images (slice thickness, 1.25 mm) were acquired from each limb using Aquilion 64TM (Toshiba Medical Systems, Otawara, Tochigi, Japan).

**Figure 1:**
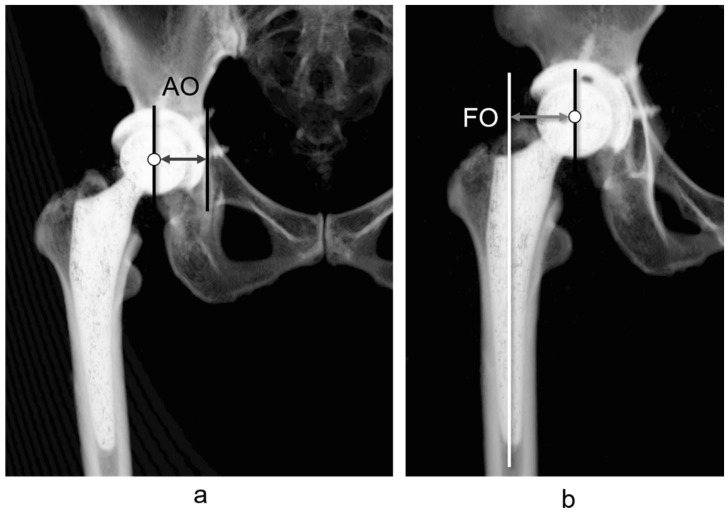
Definition of acetabular offset (AO) and femoral offset (FO). AO is the distance between the femoral head center and the most caudal point of the teardrop in the functional pelvic plane in the pelvic coordinate system (**a**). FO is the distance between the femoral head center and the anatomical axis of the femur in the femoral coordinate system (**b**).

**Figure 2:**
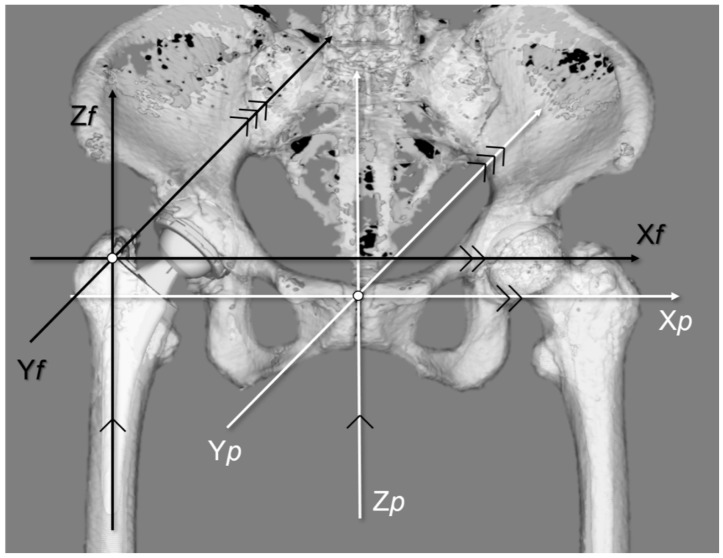
Three-dimensional parallel coordinate system ZedView^®^ (version 16.0; Lexi, Tokyo, Japan) with APP and RCP was adjusted to be parallel. The pelvis and femur X-, Y-, and Z-coordinates were parallel. APP, anterior pelvic plane; RCP, retrocondylar plane.

**Figure 3:**
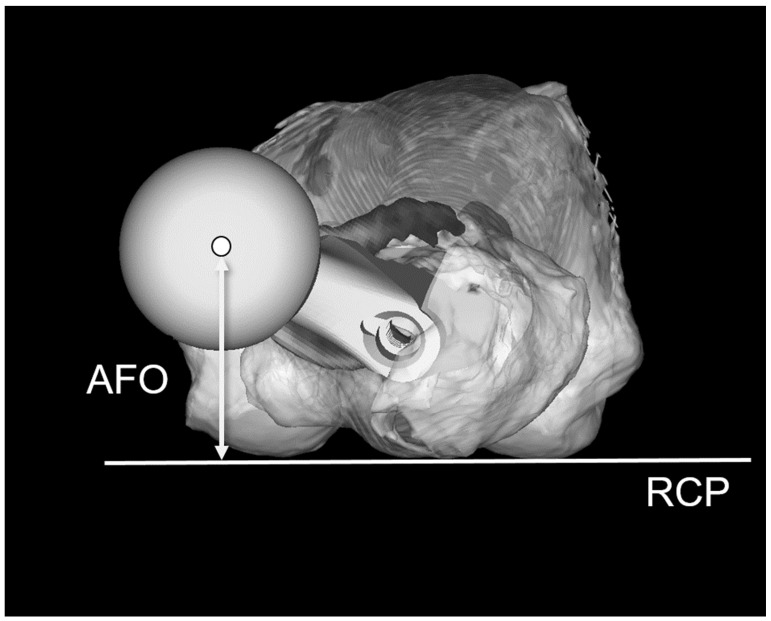
Definition of the anterior femoral offset (AFO). AFO is the distance between the femoral head center and the retrocondylar plane (RCP) in the axial plane.

**Figure 4:**
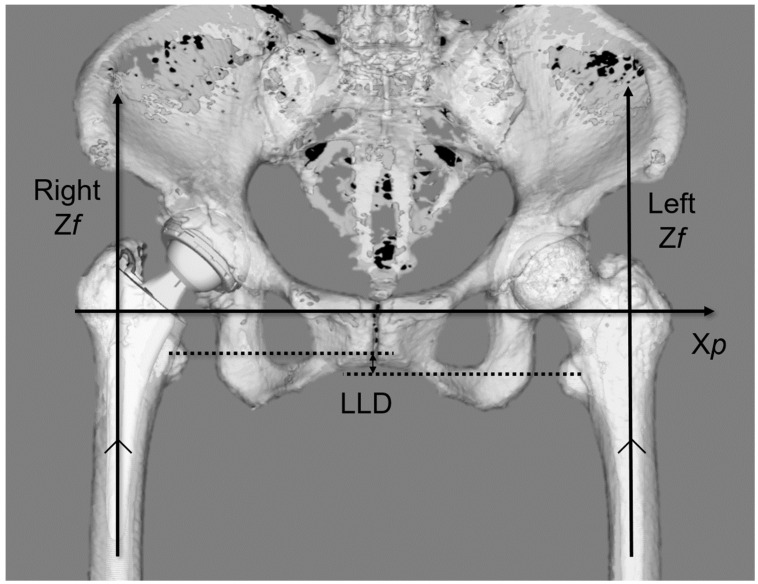
The LLD measurement. LLD is the difference in the height of the tip of the lesser trochanter between the surgical and non-surgical sides. LLD, leg length discrepancy.

First, the 3D pelvic model was adjusted for the APP. AO was defined as the distance between the teardrop and the femoral head center (Figure 1a). The 3D femur model was adjusted to the retrocondylar plane (RCP) as previously reported [24]. Furthermore, the pelvis and femur coordinate systems were adjusted to be parallel (Figure 2); therefore, the respective X, Y, and Z coordinate systems of the pelvis and femur were parallelized. The FO (distance between the femoral head center and femoral axis) (Figure 1b), AFO (Figure 3), GFO (sum of AO and FO) according to the previous study [25], and the distance from the teardrop line to the apex of the lesser trochanter (LLD: difference between the operated and non-operated sides) (Figure 4) were independent of the rotation of the pelvis and hip joint.

Moreover, we evaluated the sum of the difference in GFO between the surgical and non-surgical sides and LLD (SGL) based on a previous report [11]; the SGL was unaffected by the pelvis and hip joint rotation. These values were adjusted to 100 cm body height (100 cm BH). The angle of cup orientation was measured according to the radiographic definition [17] of the relative FPP, and the stem anteversion was measured as the angle between the stem axis and RCP in the axial plane in the femoral coordinate system. A trained orthopedic surgeon evaluated the modified HHS (mHHS) within 1 month pre-operatively and 1 year postoperatively to focus more on function and activity [26].

### 2.3. Statistical Analysis

Data were processed using IBM SPSS^®^ Statistics for Windows, version 28 (IBM Corp., Armonk, NY, USA). Linear regressions between GFO, LLD, SGL, LLD, and total mHHS scores at 1 year postoperatively were analyzed for the difference between the operated and non-operated sides since GFO and LLD are not relatively large or small [1,6]. The GFO, LLD, and SGL indices were also evaluated using binomial approximation, and they were significant according to previous studies [6]. The maximum association value was then determined.

A receiver operating characteristic (ROC) curve, which consists of absolute values around the maximum value in the binomial approximation as the center among the significant association parameters, was used to calculate a cutoff value of mHHS ≥ 80 [27] and an acceptable range. For the correlation analysis, we performed power analysis (type II (β) error) using post hoc analysis, with 0.5 and 0.05 as the effect size (d) and type I (α) error, respectively. Intra- and inter-observer reliabilities were evaluated using the intra-class correlation coefficient (ICC). Furthermore, intra-observer reliability was measured at least two times with an interval of at least 2 weeks. Statistical significance was set at *p* < 0.05.

## 3. Results

**Figure 5:**
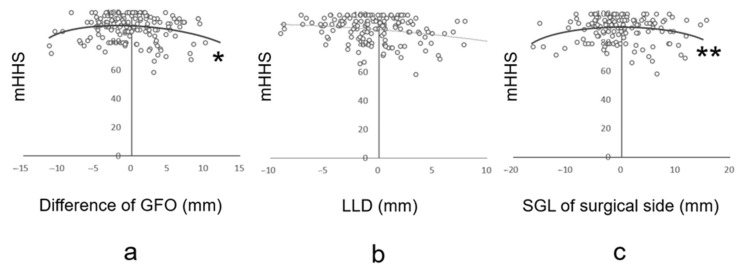
Binomial approximation and maximum value. A binomial approximation was used to determine the maximum value of any significant association. (**a**) Correlation between GFP and mHHS. (**b**) Correlation between LLD and mHHS. (**c**) Correlation between SGL and mHHS. * and ** were significantly correlated. GFO, global femoral offset; LLD, leg length discrepancy; mHHS, modified Harris Hip Score; SGL, the sum of the differences between GFO and LLD.

**Figure 6:**
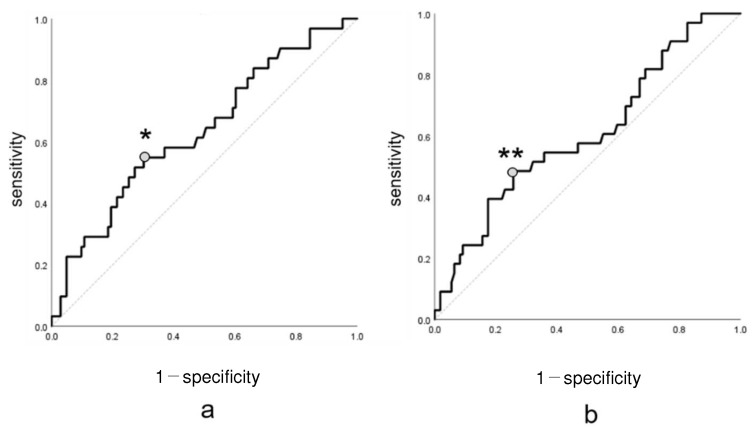
Receiver operating characteristic (ROC) curve. The cutoff value of mHHS ≥ 80 was calculated using ROC for GFO (**a**) and SGL (**b**). * and ** were the cutoff value. mHHS, modified Harris Hip Score.

Table 1 presents the participants’ details. The implantation angles were 40.0 ± 4.9°, 16.8 ± 6.1°, and 26.3 ± 10.9° for X-ray tilt, X-ray anteversion, and stem anteversion, respectively. Additionally, the mHHS scores significantly improved from 49.0 to 88.8 for all mHHS (*p* < 0.05). Table 2 presents the results. A significant binomial correlation was found between the difference in the GFO, SGL, and mHHS scores, with maximum values of 1.01 and 0.18 mm/100 cm BH (Figure 5a,c). The formulae used are listed in Table 3. An mHHS of ≥ 80 using the ROC curves had a cutoff value of 2.66 mm for GFO (area under the curve of 0.633, *p*-value of <0.025, sensitivity of 0.548, and 1-specificity of 0.301) and 4.96 mm for SGL (area under the curve of 0.616, *p*-value of 0.048, sensitivity of 0.485, and 1-specificity of 0.257) (Figure 6a,b). During the study period, no dislocations, early loosening, or revision cases occurred. Paired *t*-tests and power analyses of correlations yielded power values of 0.941 and 0.980, respectively. The ICCs for all measurements had intra- and inter-observer reliabilities of 0.828 to 0.932, respectively (Table 4).

## 4. Discussion

The major finding of this study is that the GFO should be 1 mm/100 cm, and the SGL should be adjusted to be approximately equal to improve postoperative outcomes after THA. Consequently, when the patient’s BH was 157 cm, which is our study’s mean height, the suitable values for the GFO and SGL were −2.59 to 5.76 and −7.50 to 8.07 mm, respectively. Previous studies have shown that the optimal difference between the GFO and the intact sides is approximately 5 mm [1,6]. Abduction muscle strength has been speculated to be reduced if the GFO is relatively short compared with the non-surgical side; consequently, durability, such as walking distance and speed, is reduced, resulting in the patient experiencing pain associated with muscle fatigue around the hip [28]. Conversely, if the GFO is relatively long, it may result in pain at the outer side of the hip, known as the greater trochanteric pain syndrome [6,11]. Based on our results, the suitable range of the GFO was similar to that in previous studies, i.e., −5 mm to +5 mm compared to the non-surgical side that used in 2D [1,2,3,4] and 1.2 mm/100 cm BH in 3D analyses [6]. The maximum SGL value was 0.18, which was considered optimal when the SGL value was approximately equal to that of the non-surgical side; however, the difference in LLD did not correlate with the mHHS score. According to our findings, a GFO of 1.01 and an SGL of 0.18 mm/100 cm BH longer than that of the non-surgical side leads to improved postoperative outcomes; consequently, LLD should be adjusted slightly shorter than that of the non-surgical side. Kawakami et al. [8] stated that when the perceived LLD was longer, postoperative outcomes would likely worsen, even if radiological LLDs were equal. Therefore, our findings are consistent with their results.

We performed all THAs using the acetabular component-first technique, and subsequently, the femoral component was placed; however, broaching the femur before preparing the acetabulum, also known as the “combined anteversion technique”, was reported to contribute to reducing dislocation [21,29,30]. To the best of our knowledge, we considered that if the acetabular component was placed accurately, the dislocation rate did not markedly increase even when THA was performed using the acetabular component-first technique [14,15,16,19]. No dislocations occurred during our study period. In contrast, the adjustment of GFO, LLD, and SGL depends on the depth and/or neck-shaft angle of the femoral component. Therefore, the acetabular component-first technique may be a suitable approach to adjust the offset, LLD, and SGL according to the depth and neck-shaft angle of the femoral components since it may be difficult to adjust the craniocaudal height of the acetabular component.

The findings of this study may be valuable for improving the prognosis after THA. Furthermore, surgeons should prioritize securing the GFO and SGL over the LLD when planning THA implantation, including stem anteversion. Notably, our study findings were evaluated in the pelvis and femur and were realigned to a unified coordinate system. Similarly, several studies have stated that their parameters were measured using plain radiographs; therefore, FO is not accurately influenced by differences in the rotation of the hip joint [5,6,31]. Additionally, hip adduction and abduction also influence LLD and SGL. According to Tone et al. [31], directly translating the findings obtained from plain radiographs into pre-operative 3D planning is unsuitable, particularly for stem anteversion, which may affect the offset. Therefore, this study provides a valuable report that considers and evaluates these factors, and the results may have great clinical implications.

This study had some limitations. First, only 172 patients were enrolled, approximately three times as many females as males. Pain (such as the greater trochanteric syndrome), which is likely observed in middle-aged females [32,33], may occur differently in males and females. Second, this was a retrospective cross-sectional study with short-term results. Third, the mHHS was used as this study’s assessment method. The results revealed that functional scores were associated with all of the studied parameters. Therefore, additional studies are needed to assess patient-reported outcome measures, such as the Western Ontario and McMaster Universities Osteoarthritis Index and the 12-Item Short-Form Health Survey. Using clinical scores instead of mHHS for evaluation may have further limited the optimal GFO and SGL ranges. Finally, for LLD, we evaluated the adjusted system with the X-, Y-, and Z-axes adjusted to be parallel. Recent reports have focused on “perceived LLD [8]”, and our findings were examined as “radiological LLD”. Therefore, different findings may be observed regarding “perceived LLD”. First, the surgeon should initially perform accurate pre-operative 3D planning according to our study results. Even without a navigation or robotic system, the use of fluoroscopy may improve postoperative outcomes by reproducing GFO and LLD close to those of the pre-operative plan [34,35]. Therefore, we considered that adopting our findings to perform THA in the supine position, such as the anterolateral supine or direct anterior approach, is more reasonable to confirm GFO, LLD, and SGL using intra-operative fluoroscopy [16]. Despite these limitations, to our knowledge, this is the first report to use the 3D method to measure these parameters and obtain optimal GFO and SGL. Furthermore, our results indicate that this method may improve outcomes after THA.

## 5. Conclusions

The optimal GFO and SGL were 1.01 and 0.18 mm/100 cm BH, respectively, and the optimal ranges for GFO and SGL were −1.65 to 3.67 and −4.78 to 5.14 mm/100 cm, respectively.

Our findings were assessed by realigning the pelvis and femur using a unified coordinate system. Therefore, the results of this study can be directly applied to 3D planning.

## Figures and Tables

**Table 1:** Participants’ details.

Sex (male/female)	41/131
Age (years) *	61.3 ± 10.4 (30–80)
Surgical side (right/left)	93/79
Primary disease	DDH: 129 Idiopathic osteonecrosis of the femoral head: 32 Rapidly distractive arthropathy: 6 Primary HOA: 5

* Mean ± standard deviation (range). DDH, developmental dysplasia of the hip; HOA, hip osteoarthritis.

**Table 2:** Measurement values of pelvic and femoral parameters after THA.

	Surgical Side	Non-Surgical Side	Difference between the Surgical and Non-Surgical Sides
AO (mm) *	18.7 ± 2.4 (11.7–25.7) ^†^	20.0 ± 1.8 (14.2–24.8) ^†^	−1.3 ± 2.6 (−7.3 to 5.6)
FO (mm) *	22.0 ± 3.9 (11.5–32.3) ^†^	21.0 ± 3.6 (10.6–29.1) ^†^	1.0 ± 3.6 (−8.5 to 10.4)
GFO (mm) *	40.7 ± 4.8 (27.8–52.4)	41.0 ± 4.3 (25.0–50.6)	0.3 ± 4.2 (−11.4 to 10.2)
AFO (mm) *	23.0 ± 4.7 (10.5–36.7)	22.10 ± 4.3 (9.0–36.2)	0.9 ± 5.4 (−14.3 to 13.7)
LLD (mm) *			−0.5 ± 3.4 (−9.0 to 10.1)
SGL (mm) *			−0.3 ± 5.5 (−16.3 to 15.7)

* Mean ± standard deviation (range) adjusted to 100 cm of body height, ^†^ *p* < 0.05, AO, acetabular offset; FO, femoral offset; GFO, global femoral offset; AFO, anterior femoral offset; LLD, leg length discrepancy; SGL, the sum of the differences between GFO and LLD; THA, total hip arthroplasty.

**Table 3:** Regression equation formula.

	Formula	Correlation Coefficient
*	y = −0.0726x^2^ + 0.1442x + 90.673	*r* = 0.221
**	y = −0.0298x^2^ + 0.0110x + 90.096	*r* = 0.207

**Table 4:** Reliability of the measurement values.

	Intra-Observer Reliability		Inter-Observer Reliability	
	Mean Absolute Error	ICC	Mean Absolute Error	ICC
AO (mm)	1.7 ± 1.2 (0.1–3.9) ^†^	0.915, <0.001 (0.879–0.940) *	2.0 ± 1.2 (0.1–4.2) ^†^	0.881, <0.001 (0.829–0.916) *
FO (mm)	2.1 ± 1.2 (0.2–3.9) ^†^	0.872, <0.001 (0.815–0.918) *	2.6 ± 1.3 (0.2–4.9) ^†^	0.848, <0.001 (0.792–0.897 *
GFO (mm)	2.5 ± 156 (0.0–5.1) ^†^	0.844, <0.001 (0.638–0.932) *	2.9 ± 1.8 (0.0–6.4) ^†^	0.828, <0.001 (0.624–0.923) *
AFO (mm)	1.5 ± 1.1 (0.1–3.4) ^†^	0.932, <0.001 (0.902–0.952) *	1.8 ± 1.2 (0.1–4.0) ^†^	0.889, <0.001 (0.843–0.921) *
LLD (mm)	2.0 ± 1.5 (0.0–5.6) ^†^	0.884, <0.001 (0.852–0.916) *	2.7 ± 1.6 (0.0–6.7) ^†^	0.857, <0.001 (0.810–0.899) *

^†^ Mean ± standard deviation (range), * ICC, *p*-value (95% confidential distance), ICC, intra-class correlation coefficient; AO, acetabular offset; FO, femoral offset; GFO, global femoral offset; AFO, anterior femoral offset; LLD, leg length discrepancy.

## Data Availability

Data are contained within the article.
